# In Vitro and In Vivo Studies Identify Important Features of Dengue Virus pr-E Protein Interactions

**DOI:** 10.1371/journal.ppat.1001157

**Published:** 2010-10-21

**Authors:** Aihua Zheng, Mahadevaiah Umashankar, Margaret Kielian

**Affiliations:** Department of Cell Biology, Albert Einstein College of Medicine, Bronx, New York, United States of America; Washington University School of Medicine, United States of America

## Abstract

Flaviviruses bud into the endoplasmic reticulum and are transported through the secretory pathway, where the mildly acidic environment triggers particle rearrangement and allows furin processing of the prM protein to pr and M. The peripheral pr peptide remains bound to virus at low pH and inhibits virus-membrane interaction. Upon exocytosis, the release of pr at neutral pH completes virus maturation to an infectious particle. Together this evidence suggests that pr may shield the flavivirus fusion protein E from the low pH environment of the exocytic pathway. Here we developed an in vitro system to reconstitute the interaction of dengue virus (DENV) pr with soluble truncated E proteins. At low pH recombinant pr bound to both monomeric and dimeric forms of E and blocked their membrane insertion. Exogenous pr interacted with mature infectious DENV and specifically inhibited virus fusion and infection. Alanine substitution of E H244, a highly conserved histidine residue in the pr-E interface, blocked pr-E interaction and reduced release of DENV virus-like particles. Folding, membrane insertion and trimerization of the H244A mutant E protein were preserved, and particle release could be partially rescued by neutralization of the low pH of the secretory pathway. Thus, pr acts to silence flavivirus fusion activity during virus secretion, and this function can be separated from the chaperone activity of prM. The sequence conservation of key residues involved in the flavivirus pr-E interaction suggests that this protein-protein interface may be a useful target for broad-spectrum inhibitors.

## Author Summary

Enveloped viruses infect cells by fusing their membrane with that of the host cell. Dengue virus (DENV) is an important human pathogen whose membrane fusion is triggered by low pH during virus entry into the cell. However, newly synthesized DENV must also transit through a low pH environment during virus exit. DENV is believed to escape premature fusion in the exit pathway via the small viral protein pr, which is processed and associates with virus after biosynthesis, and is released from the virus particle in the neutral pH extracellular environment. Here we have reconstituted the interaction of pr with the DENV fusion protein E using soluble protein components. The interaction has a low pH optimum and inhibits membrane insertion of the fusion protein. The recombinant pr peptide can “add back” to fully infectious mature DENV and block virus fusion and infection. We found that mutation of a critical conserved histidine on the fusion protein inhibits the interaction of E and pr, and makes the virus susceptible to low pH-induced inactivation during exit. This work characterizes the mechanism of pr protection, and suggests that the conserved multifunctional pr-E interaction may be an important target for anti-viral strategies.

## Introduction

The emergence and resurgence of human viral pathogens can be traced to a complex variety of causes including increased urbanization, human contact with animal reservoirs, a decrease in effective public health systems, and the spread of insect vectors that disseminate some viral infections [1], [2], [3]. Flaviviruses are a genus in the Flaviviridae family and include important emerging and resurgent human pathogens such as dengue virus (DENV), West Nile virus (WNV), tick-borne encephalitis virus (TBEV) and yellow fever virus [2], [4]. Flaviviruses are transmitted by insects such as mosquitoes and ticks, and can cause severe human diseases characterized by encephalitis, meningitis, and hemorrhages [2], [3]. More than one third of the world's population lives in dengue fever endemic areas, and there are an estimated 50–100 million cases of dengue infection and 500,000 cases of the more lethal complication, dengue hemorrhagic fever, per year [5], [6], [7], [8]. There are currently no antiviral therapies for flaviviruses. DENV vaccine development is underway but is problematic due to the presence of four DENV serotypes and the potential for antibody-dependent enhancement of infection [2], [6], [9], [10]. Antiviral therapies could thus be an important alternative for DENV and for viruses such as WNV in which the cost and potential side effects of vaccination must be weighed against the relatively low number of human cases [2].

**Figure 1:**
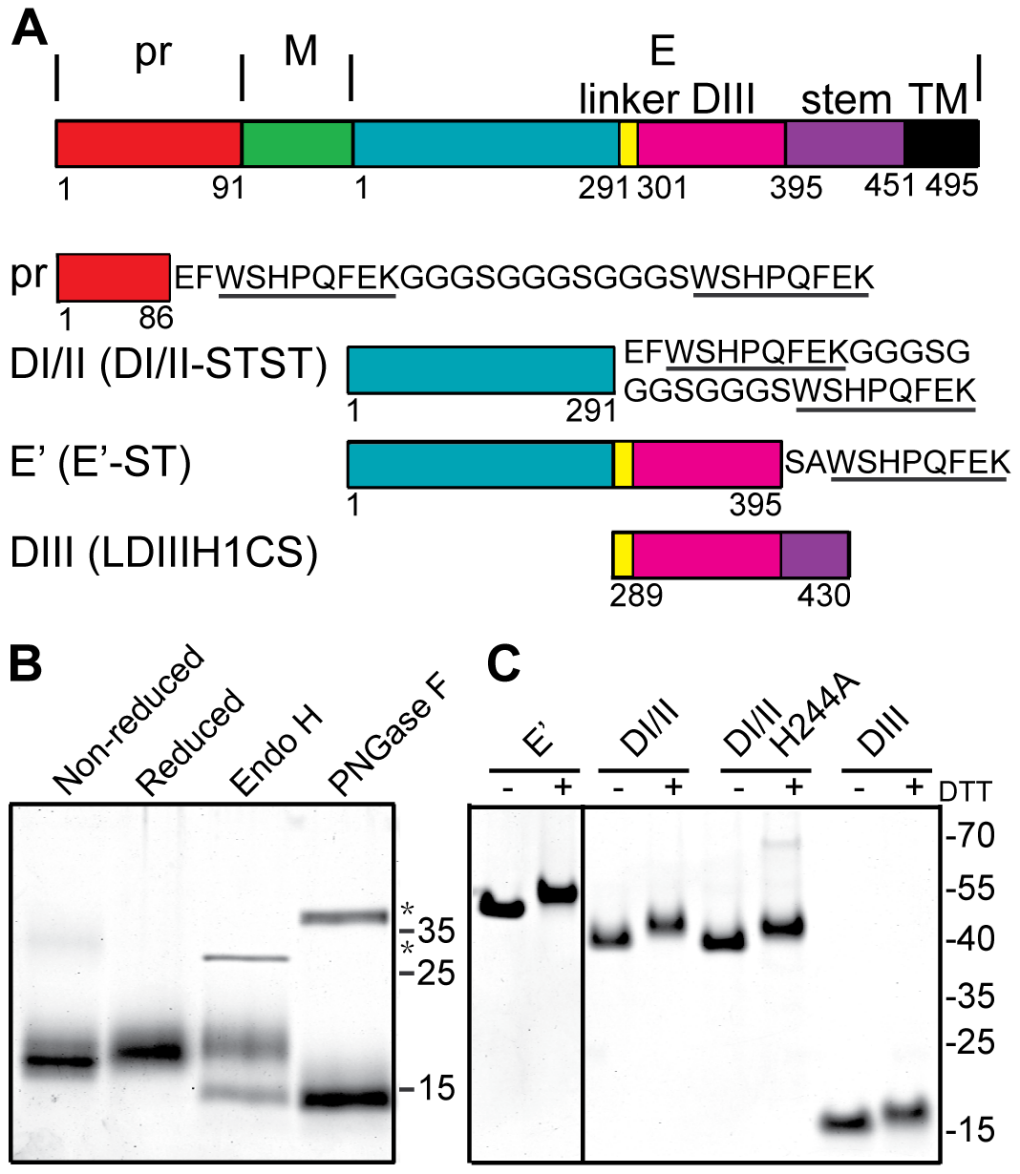
Expression and purification of DENV2 pr and truncated E proteins. A) Linear diagrams of the DENV2 prM-E proteins and the truncated DENV2 pr and E proteins used in this work (not to scale). Domain and construct boundaries are marked, with numbering based on the individual proteins in the DENV2 New Guinea C (NGC) strain. The sequences appended to the diagrams contain the *Strep* (ST) affinity tag(s) used for protein purification (underlined), joined in the case of two *Strep* tags by a flexible linker region (STST). Pr was expressed in 293T cells and contains prM residues 1–86 plus N-terminal GS residues from the vector and the STST tag. The DI/II and E′ proteins were expressed in S2 cells and contain E residues 1–291 and 1–395, respectively, plus ST or STST tags. DIII was expressed in *E. coli* and contains E residues 289–430, comprising the linker, DIII, helix 1 and conserved sequence (LDIIIH1CS). The names in parentheses are the detailed nomenclature from [30]. B) 4 µg samples of purified pr peptide were incubated with DTT, Endo H, or PNGase F as indicated, analyzed by SDS-PAGE and stained with Coomassie blue. The positions of marker proteins are shown on the right with their molecular masses listed in kilodaltons. Asterisks indicate the positions of the added glycosidases. C) 4 µg samples of purified truncated E proteins were reduced with DTT as indicated, analyzed by SDS-PAGE and stained with Coomassie blue. Marker proteins are shown on the right with their molecular masses listed in kilodaltons.

Flaviviruses are small, highly organized enveloped viruses with a spherical shape [4], [11]. They contain a positive-sense RNA genome packaged by the viral capsid protein. The nucleocapsid is surrounded by a lipid bilayer containing the viral membrane protein E. Flaviviruses infect cells by receptor engagement at the plasma membrane, endocytic uptake, and a membrane fusion reaction triggered by the low pH of the endosome compartment [12], [13]. The viral E protein binds the receptor and drives the fusion of the viral and endosome membranes to initiate virus infection. The pre-fusion structure of the E protein ectodomain (here referred to as E′) shows that E contains three domains composed primarily of β-sheets: a central domain I (DI) connecting on one side to the elongated domain II (DII) with the hydrophobic fusion loop at its tip, and connecting via a flexible linker on the other side to the immunoglobulin-like domain III (DIII) [14], [15], [16], [17], [18], [19] (Figs. 1A, S1). Although these regions are not present in the truncated E′ ectodomain, DIII connects to a stem domain and C-terminal membrane anchor (TM). The E protein in mature infectious flavivirus is organized in homodimers that lie tangential to the virus membrane [20]. Within each dimer the E proteins interact in a head to tail fashion, with the fusion loop of each E protein hidden in a hydrophobic pocket formed by DI and DIII of the dimeric E partner.

The E protein mediates virus-membrane fusion by refolding to a hairpin-like E homotrimer with the fusion loops and TM domains at the same end [21], [22]. This reaction involves low pH-triggered dissociation of the homodimer, fusion loop insertion into the endosome membrane, formation of a core trimer composed of DI and DII, and the foldback of the DIII and stem regions towards the target membrane and their packing against the core trimer. The prefusion and postfusion conformations of the flavivirus E fusion protein are structurally and functionally similar to those of the E1 fusion protein from the alphavirus Semliki Forest virus (SFV) [23], [24], [25], and these fusion proteins are often referred to as “class II” [26], [27], [28]. In addition to the ectodomains whose trimer structures are described above, truncated fusion proteins composed of domains I and II (DI/II) can reconstitute SFV and DENV core trimer formation on target membranes [29], [30]. Such core trimers act as specific targets for DIII binding, thus recapitulating the protein-protein interactions during class II trimerization and hairpin formation.

Flaviviruses bud into the endoplasmic reticulum (ER) and are transported as virus particles through the secretory pathway and released by exocytosis [4]. Given the low pH that is present in the Golgi complex and trans-Golgi network (TGN) [31], how do flaviviruses avoid inactivation during their transport? The particles are assembled in the ER as immature non-infectious viruses containing heterodimers of the precursor membrane protein (prM) and E protein [4], [26], [32]. Subsequent exposure to low pH in the secretory pathway triggers a dramatic rearrangement to E homodimers and makes the prM protein accessible to furin cleavage [33], [34]. Processing of prM by cellular furin results in mature infectious virus in which E homodimers are poised to mediate fusion [33]. Important recent studies describe the structure of pr peptide in complex with E, and indicate that processed pr remains associated with the virus at low pH and can inhibit virus-membrane interaction [34], [35], [36]. Thus, pr on the virus could protect E protein from low pH in the secretory pathway.

The flavivirus prM/pr protein plays multiple roles in the virus life cycle (reviewed in [26]). prM acts as a chaperone for E protein folding [37] and associates with the tip of E [34]. prM also appears to respond to low pH to permit E rearrangement on the virus surface and allow furin access for prM processing [34], [38]. Following cleavage, the pr peptide may prevent premature virus fusion through bridging interactions that stabilize the E homodimer and thereby prevent dissociation to E monomers, a key fusion intermediate [35], [36]. To better understand these multiple roles of prM/pr, separation of its chaperone and pH-protection functions and characterization of the pr-E interaction are needed.

Here we developed a system to produce DENV pr peptide and reconstitute the pr-E interaction in vitro. At low pH pr bound to both monomeric and dimeric forms of E and blocked their membrane insertion and trimerization. Addition of exogenous pr to mature DENV particles inhibited virus fusion and infection. Mutation of a key histidine residue in the pr-E interface, E H244, reduced pr's binding and inhibitory activity, and reduced DENV secondary infection and particle production. The defect in particle production could be partially rescued by neutralization of exocytic low pH, indicating the important role of pr in protecting DENV from premature fusion during transport to the plasma membrane.

## Results

### Expression and characterization of pr peptide

A number of truncated E proteins have been successfully produced by co-expression with prM (e.g., references [30], [39]), while the pr-E structural studies were based on a secreted hybrid protein containing truncated prM linked to truncated E [34]. Previous studies indicated that full-length TBEV prM could fold correctly when expressed in the absence of E protein [37], suggesting that production of pr peptide alone might be possible. We generated a construct based on residues 1–86 of DENV2 prM, truncating pr just before the start of the furin cleavage recognition site at residue 87 (Fig. 1A). This sequence was linked to a mammalian signal peptide at the N-terminus and to an affinity tag at the C-terminus, and expressed in 293T cells. The protein was isolated in a highly purified form by affinity chromatography and gel filtration (Fig. 1B), and was recognized by mAb prM-6.1 against prM [40] (data not shown). The pr peptide migrated at a position of ∼17 kDa in reducing SDS-PAGE, in keeping with its predicted size of 13 kDa plus the presence of carbohydrate due to the glycosylation site at position 69. This carbohydrate was removed by Peptide N-glycosidase F (PNGase F) to give a peptide of the predicted size. The protein was largely resistant to Endoglyosidase H (Endo H) digestion, indicating maturation of the carbohydrate chain as the protein transited through the Golgi complex. A mobility shift was observed upon reduction of pr, in keeping with the presence of 3 disulfide bonds in the structure of pr [34].

We also produced and purified a dimeric ectodomain form of DENV2 E protein containing all three domains (E′), a monomeric form containing E domains I and II (DI/II), and E domain III (DIII) (Fig. 1A and 1C), all as previously described in detail [30], [41].

### pH-dependent binding of pr and E proteins

**Figure 2:**
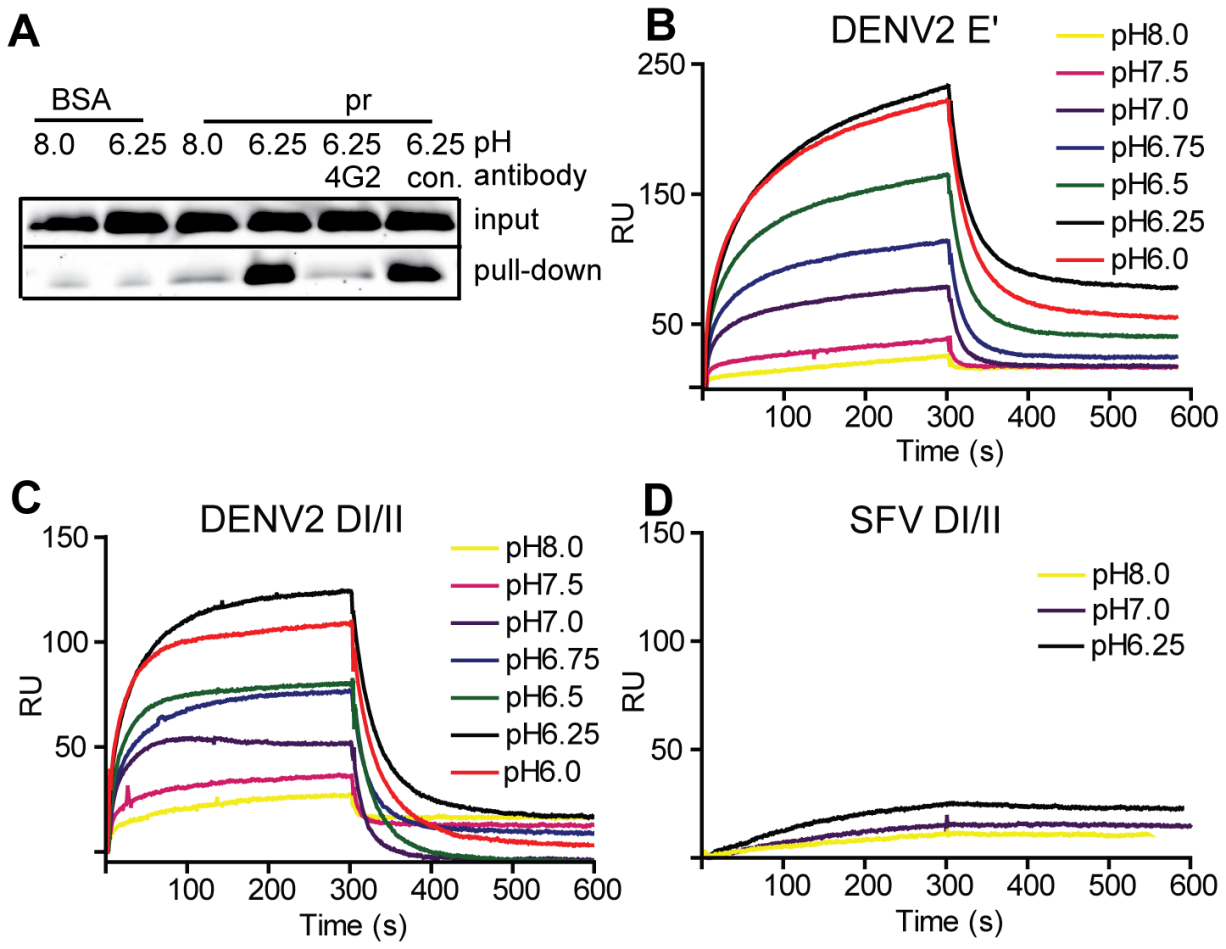
Pr peptide binds DENV E proteins in a pH-dependent manner. A) Pull-down of DI/II protein by pr. DI/II was incubated with sepharose beads conjugated with pr peptide or BSA at the indicated pH for 1 h at room temperature. As indicated, reactions contained a 2∶1 molar excess of mAb 4G2 to the E fusion loop or mAb to the ST tag (con.). Input lanes show an aliquot representing 20% of the reaction prior to pull-down. (Panels B–D) SPR analysis of pr-E binding. Pr peptide was immobilized on a CM5 sensor chip, and DENV2 E′ (B), DI/II (C) or SFV DI/II proteins (D) were flowed over the chip at concentrations of 1.2 µM in buffers of the indicated pH for 300 s, followed by injection of protein-free buffer at the same pH. Data are a representative example of two independent experiments.

As a first test of in vitro pr-E binding, we coupled pr to sepharose beads and tested its ability to pull-down truncated E protein containing only domains I and II. This form of E protein is monomeric and the tip of DII is thus accessible even at neutral pH. Previous studies showed that this and other DENV DI/II proteins are active in membrane insertion and trimerization at both neutral and low pH [30]. We observed efficient pull-down of DI/II protein by pr-sepharose (Fig. 2A), but in spite of the accessibility of the pr binding site on DI/II at neutral pH, pull-down was low pH-dependent. The pull-down of DI/II protein by pr was specific, as it was blocked by inclusion of mAb 4G2 against the E fusion loop at the DII tip, and did not occur with BSA-sepharose beads. These data suggested that the recombinant pr peptide could bind to the tip of DI/II in a low pH-dependent reaction.

For more detailed studies of pr-E binding, we performed surface plasmon resonance (SPR) assays using our various forms of recombinant E protein with immobilized pr peptide. Compared to the pull-down assay, SPR can detect low levels of protein-protein interactions as binding is detected in real time and does not require removal of unbound E. The E′ protein is a dimer at neutral pH and dissociates to monomers at low pH [30]. When SPR was performed with E′ protein buffered at pH 8.0 there was very low binding (low signal response) (Fig. 2B). As the buffer pH was decreased, the signal gradually increased, with maximal response observed at ∼pH 6.25 and no further increase at pH 6.0. A rapid decrease in signal was observed when the samples were shifted to protein-free buffer, indicating rapid dissociation of the pr-E interaction. Similar results were obtained using monomeric DI/II, with the lowest binding at pH 8.0, highest binding at pH 6.25, and a slight decrease at pH 6.0 (Fig. 2C). Thus, the dimeric E′ and monomeric E DI/II proteins bound pr peptide with similar pH-dependence. Binding to pr was specific, as little interaction was observed using the structurally similar E1 DI/II protein of SFV (Fig. 2D). In addition, binding of DENV E DI/II protein to pr was inhibited by preincubation with mAb 4G2 against the fusion loop (molar ratio 1∶1) (data not shown). Determination of the affinity of pr-E binding was not performed as the data did not fit to a simple Langmuir model of 1∶1 binding, presumably because of E protein aggregation at low pH.

### Effect of exogenous pr peptide on E protein-membrane interaction

**Figure 3:**
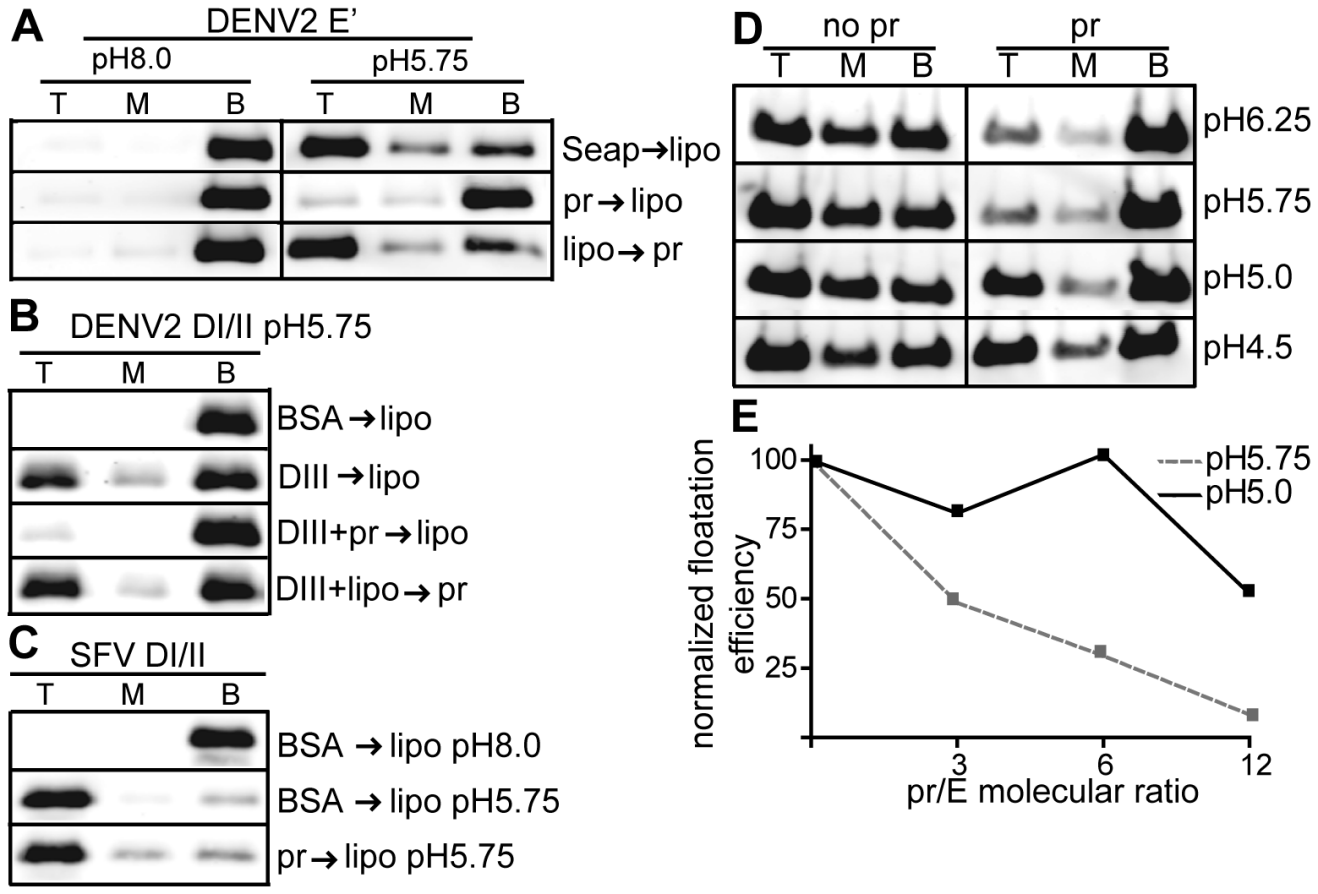
Pr peptide inhibits E protein-membrane interaction. A) E′-liposome co-floatation assay. E′ protein was mixed with pr peptide or an ST-tagged control protein (Seap) at a final concentration of 50 µg E′ protein and 200 µg pr/Seap protein/ml (molar ratio 12 pr/1E). Liposomes were added at a final concentration of 1 mM, and the samples were incubated at the indicated pH for a total of 60 min at 28°C. Where indicated, E′ protein plus liposomes were incubated for 30 min, pr peptide added to a final concentration of 200 µg/ml, and the incubation continued for an additional 30 min. The liposome-bound proteins were then separated by floatation on sucrose gradients at the indicated pH. Aliquots of the top, middle and bottom of the gradients were analyzed by SDS-PAGE and western blotting for E protein. B) DI/II-liposome co-floatation assay. 40 µg/ml DI/II plus DIII or BSA (200 µg protein/ ml) were incubated with liposomes plus 200 µg pr peptide/ml as indicated and assayed for liposome co-floatation as in panel 3A. C) SFV DI/II-liposome co-floatation assay. SFV DI/II protein (40 µg/ml) was mixed with BSA or pr peptide (160 µg/ml). Liposomes were added at a final concentration of 1 mM, and the samples were incubated at the indicated pH for a total of 30 min at 28°C. Liposome co-flotation was assayed as in panel 3A. D–E) Loss of pr inhibition of E protein in the pH range of the late endocytic pathway. D) pH dependence of pr inhibition. E′ protein was mixed with liposomes in the presence or absence of pr peptide (molar ratio ∼12 pr/1E), treated at the indicated pH as in Fig. 3A, and E′- membrane association determined by floatation assay as in Fig. 3A. E) Concentration-dependence of pr inhibition. E′ protein was mixed with liposomes and treated at pH 5.75 or pH 5.0 in the presence of the indicated molar ratios of pr peptide to E protein. E′-membrane association was determined by floatation assay as in Fig. 3A. For each pH, the E′ floatation efficiency was normalized to the amount of floatation in the top fraction in the absence of added pr protein. Data in panels A–E are each a representative example of two independent experiments.

Previous studies showed that retention of endogenous pr peptide on the furin-processed DENV particle inhibits virus interaction with liposomes at low pH [35]. Structural considerations suggested that this inhibition occurs primarily by blocking low pH-triggered dissociation of the E dimer, a required first step in the fusion reaction. To test this mechanism, we evaluated the effect of pr on the membrane interactions of dimeric and monomeric forms of E protein. The E′ dimer was preincubated with pr peptide or an unrelated protein with the same affinity tag for 5 min at pH 8.0, and then treated at pH 5.75 in the presence of target liposomes. Membrane-associated proteins were separated by liposome floatation on sucrose gradients. There was no liposome co-floatation when E′ protein was incubated with liposomes at neutral pH (Fig. 3A). About 70% of the total E′ floated with liposomes in the top part of the sucrose gradient after treatment at pH 5.75 in the presence (Fig. 3A, top panel) or absence (data not shown) of a control protein. In contrast, when E′ was preincubated with pr peptide (pr∶E′ molar ratio 12∶1) and treated with low pH, only ∼2% of E′-ST floated with the liposomes (Fig. 3A, middle panel). Inhibition by pr was not observed when it was added after E′ was treated at low pH in the presence of liposomes for 30 min (Fig. 3A, bottom panel), and thus pr needed to be present during the membrane insertion step. Inhibition was concentration-dependent, with 22% E′ co-floatation at a pr∶E′ molar ratio of 3∶1, 8% at 6∶1, and 0.4% for 24∶1 (data not shown; see also Fig. 3E).

We then tested the effect of pr on the DENV E DI/II protein. This protein is monomeric and its stable membrane interaction requires DIII to “clamp” the core trimer [30]. As shown in Fig. 3B, ∼25% of DI/II co-floated with liposomes at low pH in the present of DIII, while no co-floatation was detected when BSA was substituted for DIII protein. The addition of pr peptide blocked membrane interaction of DI/II when added prior to liposome incubation (Fig. 3B, 3^rd^ panel), but not after liposome incubation (Fig. 3B, bottom panel).

The structurally related alphavirus protein SFV E1 DI/II is monomeric and efficiently interacts with membranes at low pH (80% cofloatation, Fig. 3C, middle panel). No inhibition occurred when pr peptide was added prior to liposome addition (Fig. 3C, bottom panel), in keeping with the lack of pr-SFV DI/II binding in the SPR experiments discussed above. Thus, pr peptide specifically inhibits target membrane interaction of both monomeric and dimeric forms of the DENV E protein.

E′ protein efficiently inserted into membranes over a wide range of pH values from 6.25-4.5 (Fig. 3D–E). However, pr's inhibition of E membrane insertion was less efficient in the pH range (pH 5.0) present in the late endocytic pathway (Fig. 3D–E). This loss of pr inhibition at more acidic pH may be relevant to recent studies of infection by immature DENV [42], as mentioned in the discussion section below.

### Effect of exogenous pr peptide on dengue virus fusion and infection

**Figure 4:**
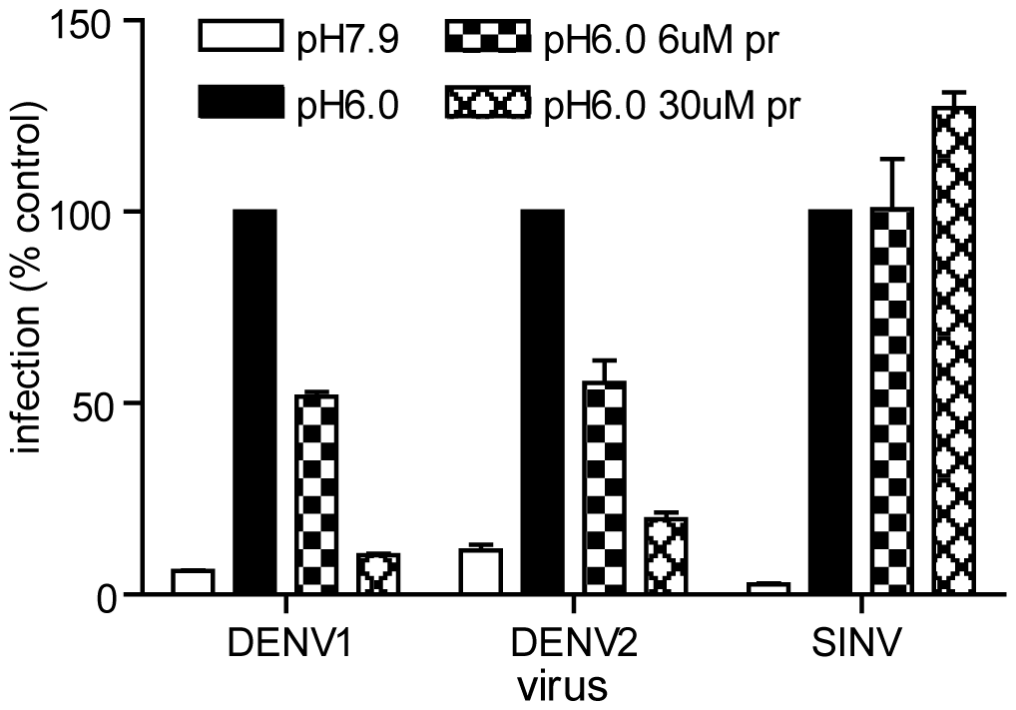
Pr peptide inhibits DENV fusion and infection. Serial dilutions of the indicated viruses were pre-bound to BHK cells by incubation for 90 min on ice at pH 7.9. The cells were then treated for 1 min at 37°C in the presence of the indicated concentration of pr peptide using buffer at pH 6.0 to trigger virus fusion with the plasma membrane, or control buffer at pH 7.9. Cells were then incubated for 48 h in the presence of NH_4_Cl to prevent secondary infection. Infected cells were quantitated by immunofluorescence, and the titers normalized to the pH 6.0 sample in the absence of pr. Each bar shows the average and range of duplicate wells. Representative example of two independent experiments.

All of the results above were obtained with soluble forms of the E protein. In order to test the ability of exogenous pr peptide to interact with and inhibit intact DENV, we took advantage of a previously described assay that monitors low pH-triggered fusion of DENV with cells [41]. In this fusion-infection assay, virus is pre-bound to target cells on ice, and then treated at 37°C for 1 min at low pH to trigger virus fusion with the plasma membrane. This fusion reaction is then quantitated by detecting the infected cells by immunofluorescence. We tested the effect of pr peptide during this 1 min low pH treatment using DENV1 WP and DENV2 NGC. The sequence of E DI/II is 68% identical between these two serotypes. Both serotypes showed efficient fusion and infection after treatment at pH 6.0, with about a 10-fold increase compared to samples treated at pH 7.9 (Fig. 4). The addition of pr peptide during the 1 min low pH treatment strongly inhibited DENV fusion and infection. Inhibition was dose-dependent, with 45–49% inhibition at 6 µM pr and 81–85-% inhibition at 30 µM pr. In contrast, pr did not inhibit low pH-triggered fusion by the alphavirus SIN (Fig. 4). Thus, exogenous DENV2 pr peptide can specifically interact with mature DENV1 and DENV2 to block virus fusion and infection. We did not observe inhibition when DENV was preincubated with 30 µM pr at pH 7.0 and then added to target cells in a standard infection assay, suggesting that under these conditions an inhibitory concentration of pr was not present during low pH-triggered fusion reaction in the endosome. This result also indicates that the presence of pr did not affect virus-cell binding.

### Role of E H244 in pr-E binding

**Figure 5:**
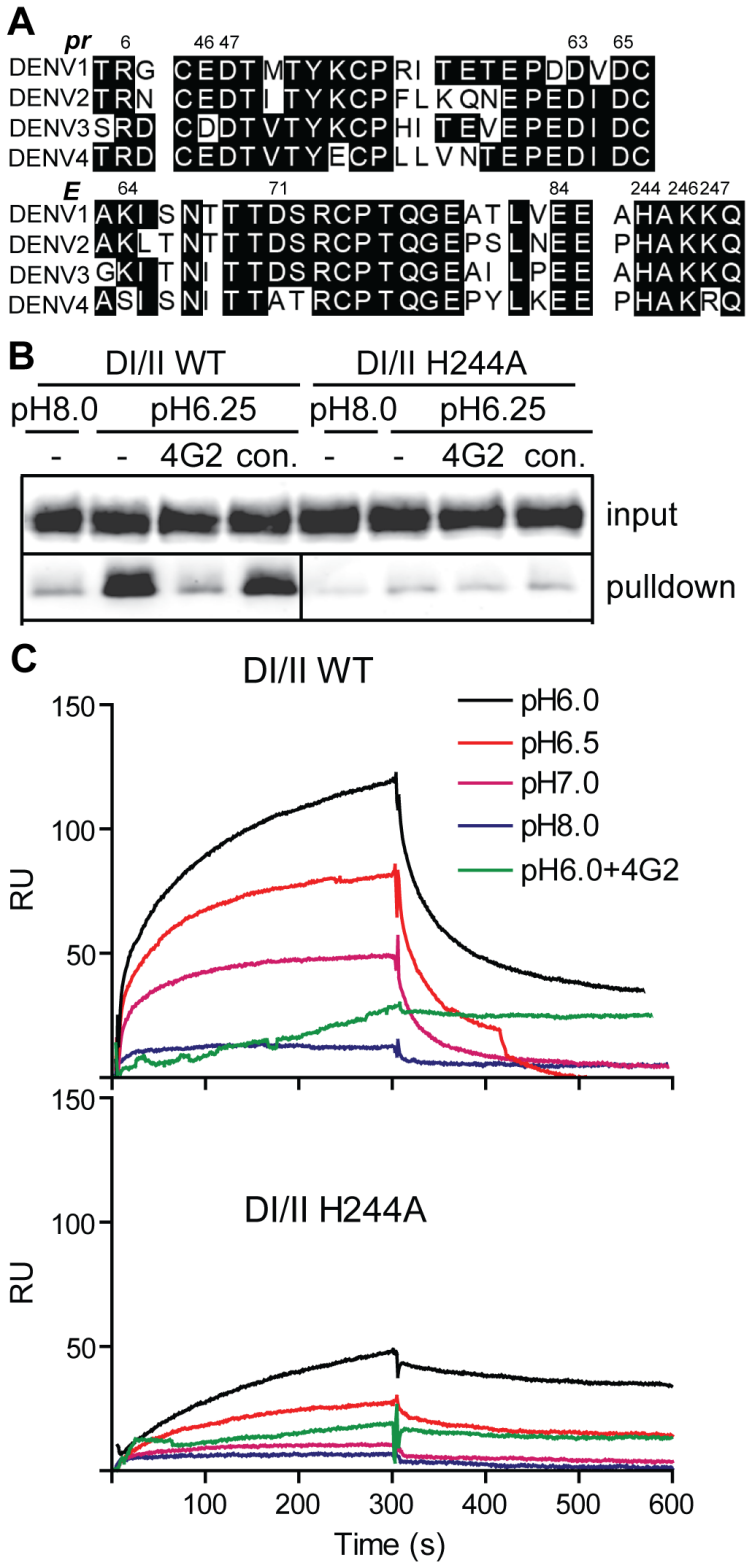
DENV E H244 is a key residue in pr-E binding. A) Sequence comparison of selected regions of the pr and E proteins from the 4 serotypes of DENV. The specific strains are DENV1 WP, DENV2 NGC, DENV3 H87 and DENV4 H241. Based on the pr-E protein structure [34], potential key residues in pr-E interaction are indicated by their numbers in the DENV2 NGC proteins. B) H244A mutation inhibits pr-E binding in pull-down assay. WT or H244A mutant forms of DI/II were assayed for binding to pr-sepharose beads as in Fig. 2A. C) H244A mutation inhibits pr-E binding in SPR assay. WT or H244A mutant forms of DI/II were assayed for binding to pr at various pH values using SPR as in Fig. 2C, shifting to buffer alone at 300 s. Where indicated, mAb 4G2 (molar ratio 1∶1) was pre-incubated 15 min at room temperature with DI/II proteins at pH 6.0 prior to assay. Data are a representative example of two independent experiments.

Although the interaction of pr with DENV can clearly prevent virus-membrane interaction and fusion (this study and [35]), the importance of pr in protecting DENV during exocytic transport has not been defined. The binding interface between prM and E contains three complementary electrostatic patches containing 11 residues [34] (see also Fig. S1). Sequence analysis shows that these 11 residues (Fig. 5A, numbered residues) are highly conserved among the 4 DENV serotypes, and that D63 and D65 of pr, and the complementary H244 on E protein are conserved among all reported flavivirus sequences [34]. Optimal pr-E binding in vitro occurred at ∼pH 6.25 (Fig. 2), suggesting that protonation of H244 could be involved in this pH-dependence. To test this we substituted alanine for H244 in the DI/II protein. DI/II H244A was produced in highly purified form with electrophoretic mobility similar to that of the wild type (WT) protein in reducing and non-reducing SDS-PAGE (Fig. 1C).

We first tested the effect of the H244A mutation on pr-E binding. In agreement with our earlier results, WT DI/II protein was efficiently pulled-down by pr-sepharose (Fig. 5B). Pull-down was low pH-dependent and blocked by mAb 4G2 against the E fusion loop at the DII tip. In contrast, almost no H244A DI/II protein was pulled-down by pr-sepharose at either low pH or neural pH (Fig. 5B). SPR analysis of WT DI/II protein showed most efficient binding at pH 6.0, and binding was blocked by pre-incubating the DI/II protein with mAb 4G2 (molar ratio 1∶1) before dilution into SPR buffer (Fig. 5C, upper panel). Equivalent concentrations of H244A DI/II protein showed greatly reduced binding to pr compared to that of WT protein (Fig. 5C, lower panel). Although H244A binding was decreased, the residual binding was still blocked by mAb 4G2 and had an acidic pH optimum. This suggests that binding also involves other residues in the pr-E interface, such as the complementary residues identified in the structural studies and shown in Fig. 5A.

**Figure 6:**
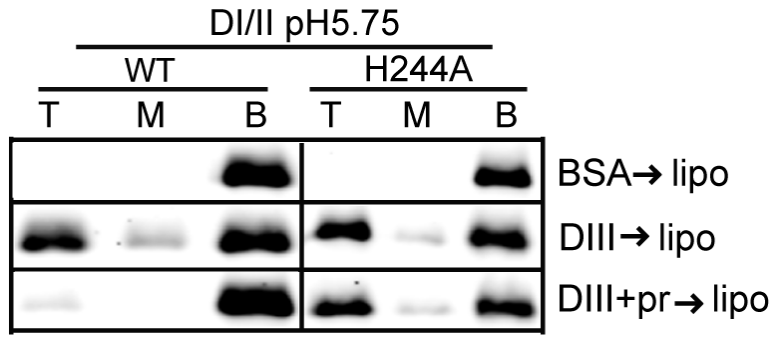
H244A E protein interacts with membranes and is resistant to inhibition by pr. WT or H244A DENV2 DI/II proteins (40 µg/ml) were mixed with DIII or BSA (200 µg/ml) in the presence or absence of pr peptide (200 µg/ml). Liposomes were added at a final concentration of 1 mM, and the samples were incubated at pH 5.75 for a total of 60 min at 28°C. Samples were analyzed by floatation on sucrose gradients at pH 5.75 as in Figure 3A. Data are a representative example of two independent experiments.

We then asked if the H244A DI/II protein was still active in binding to target liposomes. WT or mutant DI/II proteins were mixed with liposomes at low pH in the presence of DIII protein to stabilize the core trimer. Both proteins efficiently bound liposomes in a DIII-dependent reaction (Fig. 6), indicating that the mutant protein retains its ability to insert into target membranes and form a core trimer. In agreement with the results in Fig. 3C, floatation of the WT protein was blocked by inclusion of pr during the membrane insertion step (Fig. 6). In contrast, the efficiency of floatation of the H244A mutant protein was 43% in the absence of pr and 47% in the presence of pr. Thus, the H244A mutation did not inhibit E-membrane interaction but made that interaction insensitive to the presence of pr.

### H244A mutation inhibits DENV secondary infection

**Figure 7:**
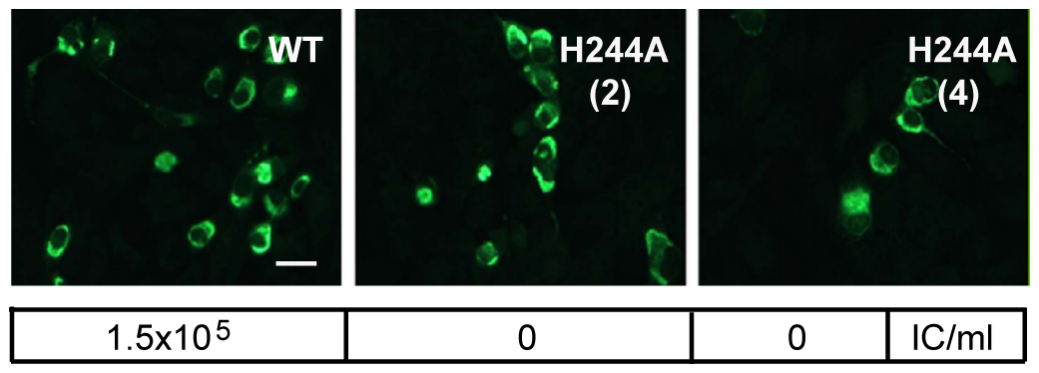
DENV E H244A mutation inhibits virus infection. RNAs derived from the WT and E H244A mutant DENV1 WP infectious clones were electroporated into BHK cells. Cells were cultured for 3 d and infected cells were detected by immunofluorescence. In parallel, cells were cultured at 28°C for 6 d and progeny virus in the culture medium was quantitated using infectious center assays on indicator BHK cells. Progeny virus titers are shown in the box below each fluorescence image. Results are given for two independent infectious clones of H244A, indicated as (2) and (4). Bar represents 30 µm.

Since the E H244A mutation disrupts E protein's interaction with pr, we used this mutation to address the importance of pr in protecting DENV during transport through the exocytic pathway. We introduced the E H244A mutation into the infectious clone of DENV1 WP. WT and mutant viral RNAs were prepared by in vitro transcription and were electroporated into BHK cells. After culture for 3 d at 37°C, both WT and mutant RNA-electroporated cells expressed abundant E protein as detected by immunofluorescence microscopy (Fig. 7). Parallel cultures were incubated for 6 d and progeny virus in the culture media was detected by infectious center assays on indicator BHK cells. WT-infected cells produced infectious progeny virus with a titer of ∼1.5×10^5^ IC/ml. However, two independent infectious clones of the H244A mutant produced no detectable progeny virus, even though the viral RNAs mediated efficient primary infection as shown in Fig. 7. This agrees with previous studies indicating lethal effects of an H244A mutation on DENV2 [43].

### Role of E H244 during virus assembly and secretion

The absence of secondary infection by the H244A DENV1 mutant could be due to decreased virus particle production and/or production of particles that are non-infectious. Efficient DENV particle production is dependent on E protein folding, particle budding into the ER, and subsequent particle egress through the secretory pathway. To investigate these issues, we took advantage of the ability of the flavivirus prM and E proteins to assemble into virus-like particles (VLP) in the absence of other viral components or virus infection [44], [45], [46]. The VLP system avoids complications arising from selection of revertants of deleterious virus mutations such as H244A. Flavivirus VLP bud into the ER in the immature prM form, undergo furin maturation during transport through the secretory pathway, and display similar low pH-dependent fusion activity as infectious virions [44], [47]. The VLP system has been used extensively to follow the process of flavivirus particle production and the role of prM in this process [37], [44], [45], [48].

**Figure 8:**
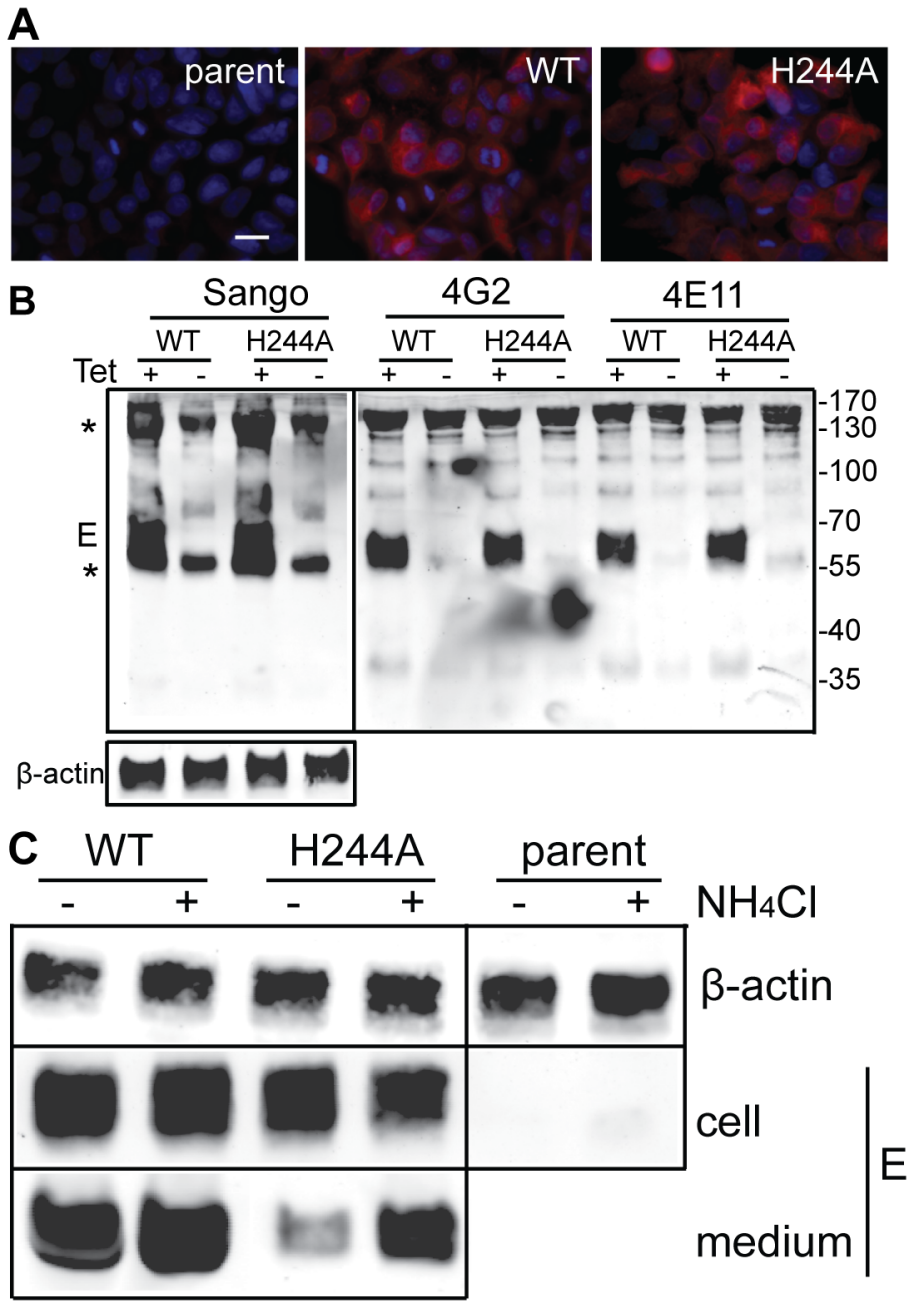
DENV E H244A mutation inhibits release of virus-like particles via a low pH-dependent mechanism. A) WT and H244A mutant E proteins are comparably expressed. Stable cells inducibly expressing the WT or H244A mutant forms of prM-E were treated with tetracycline for 36 h at 37°C. E protein expression was detected by immunofluorescence and the nuclei were stained with DAPI. Fluorescence images are shown at the same magnification and exposure time. Bar represents 30 µm. B) WT and H244A mutant E proteins are comparably immunoprecipitated by conformation-specific mAbs. Stable cells inducibly expressing the WT or H244A mutant forms of prM-E were treated with tetracycline for 36 h at 37°C. E proteins in the cell lysates were immunoprecipitated by Sango, a rabbit polyclonal antibody to DIII, and by the mouse mAbs 4G2 and 4E11, as indicated at the top of the panel. Samples were then analyzed by SDS-PAGE and western blot using mouse anti-DENV2 Ab for the Sango samples and Sango for the mAbs samples. Asterisks indicate the positions of the IgG and IgG heavy chain, which cross-react in the western blot. Equivalent sample input was evaluated by western blot for β-actin (lower panel). C) Effect of low pH on WT and H244A VLP production. WT and H244A mutant cells were incubated with tetracycline for 2 h and then in this medium plus 20mM NH_4_Cl where indicated for a total of 36h. VLP released in the culture media were pelleted by ultracentrifugation, and E proteins in the cell lysates were immunoprecipitated using mAb 4G2. VLP and lysate samples were analyzed by SDS-PAGE and western blot using Sango. 5-fold more culture media from the H244A cells than the WT cells were loaded. Data are representative examples of two or more independent experiments.

We established stable HEK 293 cells that inducibly express the DENV1 WT or H244A prM-E proteins. After 36 h induction with tetracycline, both WT and H244A cells show abundant intracellular expression of the DENV1 E protein as detected by immunofluorescence, while the parent cell line is negative for E expression (Fig. 8A). To evaluate whether WT and H244A E proteins were correctly folded, cells were induced for 36 h, lysed, and immunoprecipitated with a rabbit polyclonal antibody to E DIII, and with two conformation-specific mAbs. mAb 4E11 recognizes a discontinuous epitope on DENV E DIII and requires proper DIII disulfide bond formation for recognition [49], [50]. mAb 4G2 recognizes the fusion loop at the tip of flavivirus E DII and its epitope is sensitive to reduction [51]. Expression studies have shown that the 4G2 epitope is not formed if the E protein is expressed in the absence of prM [52], indicating that this epitope is particularly useful for diagnostic tests of prM's chaperone interaction with E (see also reference [37]). As shown in Fig. 8B, lysates from cells induced to express prM plus WT or H244A E proteins showed strong reactivity with all three antibodies. Quantitation of multiple experiments confirmed that WT and H244A E proteins were comparably recognized by the 4E11 and 4G2 mAbs. Thus, by these criteria H244A E protein interacts with prM protein and is correctly folded. This result also agrees with our finding that truncated H244A E protein expressed with prM in the S2 cell system was fully active in low pH-dependent membrane binding and trimerization, suggesting correct folding (Fig. 6).

We then used the inducible cells to examine VLP production. Expression was induced for 36 h. The cells were then lysed and the E proteins immunoprecipitated, and the VLP in the culture media were pelleted by ultracentrifugation. Analysis by western blotting showed strong E protein expression in both WT and H244A cells, and no expression in the parent cells (Fig. 8C). The WT cells released E protein in VLP, but VLP release from cells expressing the H244A mutant E protein was greatly reduced (Fig. 8C, - media samples). This result is in keeping with the hypothesis that the H244A cells assemble VLP in the neutral pH environment of the ER but that VLP release is inhibited by the lack of pr protection from the low pH of the secretory pathway. To test this idea, we induced WT and H244A prM-E expression and cultured the cells in the presence of 20 mM NH_4_Cl to neutralize the acidic pH in the Golgi and TGN compartments (Fig. 8C, +NH_4_Cl lanes). The cellular expression level of either E protein was not significantly affected by NH_4_Cl treatment, and WT VLP production was similar in NH_4_Cl-treated cells and untreated cells. However, production of VLP containing the H244A mutant E protein was increased 4–7 fold in NH_4_Cl-treated cells. While H244A VLP production was still significantly decreased compared to that of WT, it was selectively rescued by NH_4_Cl treatment.

## Discussion

During translation of the flavivirus polyprotein, prM is the first protein translocated into the ER lumen, where it acts as a chaperone during the folding of the subsequently translocated E protein [4], [37], [44]. In addition to this important role of prM during E protein synthesis, a variety of data suggest that the interaction of pr peptide with the viral E protein protects flaviviruses from low pH during their transport through the exocytic pathway [34], [35], [36]. Here we showed that a recombinant pr peptide was efficiently folded, glycosylated, and secreted from 293T cells in the absence of its normal prM context and furin processing. Recombinant pr bound to soluble E proteins at low pH, inhibited E-membrane insertion, and interacted with mature dengue virus to block fusion and infection. Alanine substitution of the conserved E H244 within the pr-E interface disrupted pr-E binding in vitro and blocked secondary virus infection. VLP production was inhibited by the H244A mutation and partially rescued by pH neutralization with NH_4_Cl. Together our data demonstrate the critical role of pr in protecting DENV from exocytic low pH.

### Properties of pr-E binding

The in vitro interaction of pr with various truncated forms of E protein was strongly pH-dependent, with a pH optimum of ∼6.25. In situ measurements indicate that the pH of the TGN is ∼6 [53], while the pH optimum of DENV2 NGC fusion is ∼6.2 [41]. The low pH of the TGN is critical for the rearrangement of immature DENV to allow furin cleavage, but once the virus is processed it becomes fusion-active in this same pH range. Thus the pH dependence of the pr-E interaction appears optimized to protect DENV during its continued transit through the secretory pathway. Pr's inhibition of E membrane insertion was less efficient at a pH value (pH 5.0) similar to that in the late endocytic pathway (Fig. 3D–E). This loss of pr inhibition at more acidic pH could help to explain the recent finding that infection by immature DENV is enhanced by antibodies to prM [42]. The antibody-bound immature virus is likely to be endocytosed and processed by cellular furin in the endocytic pathway [54]. The lower pH conditions of the late endocytic pathway could then cause the loss of pr inhibition and allow virus fusion.

The structure of furin-cleaved DENV at pH 6.0 shows that pr is bound to the virion through interactions with the DII tip of one E protein and DI on the neighboring E monomer [35], [36]. This suggested that pr might primarily block virus-membrane interaction by preventing dissociation of E dimers, a required first step in the fusion pathway [55]. Our results show efficient binding of pr to the dimeric form of the DENV E protein, but also to the monomeric DI/II form. We do not know if the E′ protein dimer is stabilized by pr interaction or if the dimer dissociates prior to interaction with pr, and experiments to address these points were inconclusive (data not shown). The similar pH dependence of pr binding to monomeric and dimeric E proteins suggests that pr may bind the same site in both cases. mAb 4G2 against the fusion loop inhibited pr interaction with E DI/II, confirming that pr was binding to the DII tip rather than to other sites on expressed E proteins. In keeping with its binding site in the vicinity of the fusion loop, pr peptide blocked the membrane insertion and liposome co-floatation of E′ and DI/II proteins. Prior studies showed that a monomeric DI/II protein with a single *Strep* affinity tag stably inserts into liposomes at either neutral or low pH [30], and pr blocked this insertion even at pH 8.0 where its interaction with DI/II was suboptimal (data not shown). Thus, while the pr-E interaction is strongly low pH-dependent, its functional inhibition of membrane insertion can still be observed at neutral pH in the presence of excess pr.

### Effects of E protein H244 mutations

Several other studies have addressed the role of E H244 in the flavivirus lifecycle. Experiments in TBEV evaluated particle production and membrane fusion activity using a VLP system [56]. Mutation of H248 (TBE numbering) to A or I blocks VLP secretion, in agreement with our results. However, an H248N mutant efficiently produces VLP, and these particles show WT levels of fusion activity. WNV E H246A or Q mutations inhibit release of infectious reporter virus particles from cells, as do a number of other substitutions at this position [57]. Replacement of H246 with aromatic residues such as phenylalanine allows both particle release and infectivity. An H244A mutation in DENV2 NGC inhibits infectious virus production [43]. E H244 and its interacting partners D63 and D65 on pr are conserved within the flaviviruses, and thus these data from several flaviviruses plus our DENV results support an important role for the E 244 position. However, a histidine residue at this position does not seem to be strictly required for particle production, suggesting that substitutions such as 244F and 244N can support the interaction of E with pr.

In contrast to the block in production of H244A VLP, the H244A DI/II protein was efficiently secreted from cells. Mutant protein secretion was somewhat reduced, with the final yield of DI/II H244A about half that of the WT protein in two separate preparations (data not shown), suggesting some effects of non-optimal pr interaction. However, unlike the E protein in virus or VLP, the truncated DI/II protein lacks the TM region and does not mediate membrane fusion, and thus may be relatively independent of the pH-protection function of pr. The purified WT and mutant DI/II proteins were able to bind liposomes and form core trimers that were stabilized by DIII (Fig. 6). Thus, the mutant protein is correctly folded and active in membrane insertion. Studies with conformation-specific mAbs also provided evidence for the correct folding of H244A E protein (Fig. 8B). Together, these results suggest that the H244A E protein is still able to access the chaperone functions of prM, while its decreased pr binding indicates that it can no longer utilize the pH protection functions of pr.

These data are consistent with the idea that, similar to WT E, the mutant protein is assembled with prM into VLP in the ER. The membrane insertion and trimerization activity of H244A suggest that the full-length mutant protein would be fusion-active on such VLP once they are transported from the neutral pH of the ER to the low pH of the Golgi and TGN [31]. Thus, the decreased release of H244A VLP and its partial rescue by neutralization of the exocytic pathway support a critical role for pr in protecting DENV from exocytic low pH, and suggest that virus/VLP fuses in the TGN in the absence of pr-E interaction. Rescue of H244A VLP production by NH_4_Cl was clearly incomplete. This may be due to complex aspects of both virus and cell, such as direct effects of the H244A mutation on particle assembly in the ER, or difficulties in blocking fusion of a virus with the relatively high pH threshold of DENV.

### Implications of the in vitro pr-E interaction

Several strategies have been used to block flavivirus and alphavirus fusion reactions and thus inhibit virus infection. SFV and DENV fusion are specifically blocked by exogenous DIII, which binds to the core trimer and prevents the foldback of endogenous DIII and hairpin formation [41]. A later stage in DENV fusion is targeted by a stem-derived peptide, which binds to the ectodomain trimer in which DIII has folded back but stem packing has not yet occurred [58]. These virus protein-protein interactions can be reconstituted in vitro [29], [30], [58], opening the possibility of using them as screens for small molecule inhibitors of virus fusion and infection.

The in vitro reconstitution of the pr-E interaction using soluble components could also act as a screen for small molecule inhibitors of this important flavivirus protein-protein interaction. Such inhibitors could act at multiple points in the virus lifecycle. During virus protein biosynthesis, an inhibitor could block the chaperone interaction of prM with E, leading to misfolding of E and its elimination by the ER quality control pathway. An inhibitor of pr interaction could make E protein susceptible to premature fusion in the TGN and could thus block virus production similar to the H244A mutation. It is also possible that small molecule inhibitors of pr-E binding could interact directly with the DII tip on mature virus particles, perhaps stabilizing the dimer and/or blocking membrane insertion of the fusion loop, thereby blocking virus fusion. Thus the in vitro system we describe here has the potential to identify molecules that could aid in the study of the flavivirus lifecycle and that could act to inhibit specific steps.

### Effects of pr on virus fusion

Previous studies showed that after cleavage endogenous pr is retained on the virus particle if the virus is maintained at acidic pH [35]. Under these conditions, the virus-pr complex does not bind target membranes, while virus from which pr is first released at neutral pH efficiently binds membranes upon shift to acid pH. Thus, the bound endogenous pr inhibits virus-membrane interaction and presumably blocks virus fusion [35]. Our results demonstrated that even after maturation to fully infectious DENV particles, exogenous pr could add back to the virus and inhibit low pH-triggered virus fusion and infection. The flavivirus membrane fusion reaction is very rapid, occurring within seconds of low pH treatment [47]. Recombinant DENV2 pr peptide inhibited fusion by both DENV1 and DENV2, suggestive of a fairly broad spectrum inhibition in agreement with the strong sequence conservation at the pr-E interface [34].

The structure of the flavivirus E protein in its pre-fusion and post-fusion conformations defines the dramatic conformational changes between these two states. Many questions about the intermediates that connect the pre- and post-fusion conformations remain. In particular, it will be important to define the membrane protein rearrangements in the context of the highly organized flavivirus particle. For example, a neutralizing E mAb that blocks virus fusion was used to trap a West Nile virus fusion intermediate [59]. It will be interesting to evaluate if exogenous pr peptide could also be used as a novel probe to capture intermediates in the flavivirus fusion pathway.

## Materials and Methods

### Cells, viruses and antibodies

BHK-21 cells and C6/36 mosquito cells were cultured as described previously [60]. 293T cells and T-REx™-293 cells were cultured as previously described using tetracycline-deficient fetal calf serum for the latter cells [61]. The DENV2 New Guinea C (NGC) strain and the DENV1 Western Pacific (WP) strain were propagated in C6/36 cells in DMEM containing 2% heat-inactivated fetal calf serum and 10 mM Hepes, pH 8.0, as previously described [41], [62]. Sindbis virus expressing green fluorescent protein was obtained as an infectious clone (a kind gift from Dr. Hans Heidner) and propagated in BHK cells [63].

4G2 is a mouse monoclonal antibody (mAb) that recognizes the fusion loop of flavivirus E proteins [51], [64]. mAb prM-6.1 recognizes a linear epitope on prM, and was a kind gift of Drs. Chunya Puttikhunt and Nopporn Sittisombut [40]. 4E11 is a mouse mAb that recognizes DIII of DENV E protein and neutralizes all 4 serotypes of dengue virus [49], [50], and was a kind gift of Dr. Fernando Arenzana-Seisdedos (Institute Pasteur, Paris). The anti-DIII polyclonal antibody Sango was raised by immunization of a rabbit with purified DENV2 DIII protein [30]. Western blot detection of truncated E proteins used 4G2 or Sango antibodies. A mAb to β-actin was obtained from Sigma and used to confirm equivalent loading of cell lysate samples. Immunofluorescence detection of DENV-infected cells used the antibody to DIII or mouse polyclonal anti-DENV2 hyperimmune ascitic fluid (obtained from Robert B. Tesh, University of Texas Medical Branch), with Alexa Fluor 488 or rhodamine-conjugated secondary antibodies (Molecular Probes).

### Protein expression, purification, and quantitation

The sequence encoding residues 1–86 of pr was amplified by PCR of an expression plasmid for DENV2 NGC prM-E DI/II [30]. The PCR product was ligated into the pPUR vector (Clontech), with the 21-residue TPA signal peptide [65] fused at the N-terminus and a tandem *Strep* tag at the C terminus (Fig. 1). The plasmid, referred to as pPUR-TPA-pr-STST, was transfected into 293T cells using polyethylenimine (PEI, Polysciences). For optimal protein production, 3.5×10^6^ cells were plated per 10 cm dish and cultured for 24 h in 10 ml of complete medium. 7.5 µg plasmid in 1 ml DME was mixed with 30 µg PEI, incubated 10 min, then added drop wise to the cell culture medium. After 12 h, the medium was changed to 10 ml DME plus 2% serum. The culture medium was collected after 48h and again after 72h. Pr was purified by affinity chromatography on a *Strep*-Tactin column from IBA BioTAGnology and by gel filtration using a Sephadex G75 column [30]. Final yields were ∼2 mg purified protein/1 liter culture supernatant.

Truncated DENV E proteins (Fig. 1) were obtained by inducible expression in Drosophila S2 cells and purified by affinity chromatography as previously described in detail [29], [30]. The H244A mutation was introduced into the DI/II protein by in vitro mutagenesis, and S2 cell expression and purification were performed as above. DENV2 NGC DIII (Fig. 1) was previously referred to as LDIIIH1CS [30], and contains domain III, the linker between domain I and domain III, and the H1 and CS regions of the stem domain. DIII was expressed in *E.coli* and refolded as previously described [41]. SFV E1 DI/II protein was produced as previously described [29]. All purified proteins were stored in TAN buffer (20 mM Triethanolamine[TEA], pH 8.0; 130 mM NaCl) at −80°C.

SDS-PAGE analysis was performed using 10–12% acrylamide gels with a Bis-Tris buffer system (Invitrogen). Western blots were performed with Alexa Fluor 688-conjugated secondary antibodies (Molecular Probes), and were quantitated using an Odyssey Infrared Imaging system and Odyssey InCell Western software (LI-COR Biosciences) [30]. Standard curves with purified E proteins confirmed the linearity of this analysis (data not shown).

### Pull down assay

Pr or BSA was coupled to NHS-activated sepharose 4 fast flow (GE Healthcare) as described in the manual. In brief, sepharose was washed with 1mM HCl, and incubated with 660 µg pr or BSA/ml in 0.2 M NaHCO_3_, 0.5 M NaCl, pH 8.3 at room temperature for 1.5 hr. The reaction was quenched with 0.1M Tris-HCl pH 8.5 for 30 min and free protein removed by washing with PBS. About 1mg of protein was coupled to 1ml beads. For the pull-down assay, 3 µg DI/II protein was pre-incubated where indicated with 24 µg 4G2 (molar ratio 1∶2) or control mAb for 10 min at room temperature, and then incubated for 1 h on a rocker at room temperature with 10 µl of pr- or BSA-sepharose in a buffer containing 20 mM MES, 20 mM TEA, 130 mM NaCl, 0.2% Tween 20 at pH 8.0 or 6.25. The beads were then washed twice with the corresponding buffer and the bound DI/II was analyzed by SDS-PAGE and western blot.

### Surface plasmon resonance assays

SPR studies were performed on a BIAcore 2000 instrument (GE Healthcare). Purified recombinant pr was immobilized on a CM5 biosensor chip by primary amine coupling as described in the manual. In brief, pr peptide was diluted to 10 µg/ml in 10 mM sodium acetate pH 4.7 and pre-concentrated on the chip surface. The chip was then activated by a mixture of 1-ethyl-3-(3-dimethylaminopropyl)carbodiimide and N-hydroxysuccinimide, followed by quenching with 1M ethanolamine at pH 8.5. Under these conditions, pr was immobilized to a final density of 600 or 1000 response unit (RU). A control cell was mock-coupled with protein-free solutions. To test interaction, truncated E proteins were diluted to 1.2 mM in a MES/TEA buffer (20 mM MES, 20 mM TEA, 130 mM NaCl) at a pH range of 6.0 to 8.0, and flowed over the chip for 300 s at 0.3 µl/min, followed by buffer alone at the same flow rate. After each round, the chip was regenerated by washing with 50 mM NaOH in 1 M NaCl. The pr chip showed undiminished E binding activity for at least 50 rounds.

### Liposome floatation assay

Liposomes were prepared by freeze-thaw and extrusion through 200 nm polycarbonate filters [66], and were stored at 4°C in TAN buffer under N_2_ and used within 2 weeks of preparation. Liposomes were composed of a 1∶1∶1∶3 molar ratio of 1-palmitoyl-2-oleoyl-sn-glycero-3-phosphocholine (POPC), 1-palmitoyl-2-oleoyl-sn-glycero-3-phosphoethanolamine (POPE), sphingomyelin (bovine brain) (Avanti Polar Lipids; Alabaster, AL), and cholesterol (Steraloids, Inc.; Wilton, NH), plus trace amounts of ^3^H-cholesterol (Amersham; Arlington Heights, IL).

Protein-membrane interaction was monitored using a liposome co-floatation assay [29], [30]. E′ or DI/II proteins at a final concentration of 50 µg/ml were incubated in TAN buffer (pH 8.0) for 5 min at 28°C in the presence of 200 µg pr peptide/ml as indicated. Liposomes were then added to a final concentration of 1mM lipid and the samples were adjusted to pH 5.75 by the addition of 0.3 M MES or maintained at pH 8.0, and the incubation continued at 28°C for 30–60 min. The samples were then adjusted to 20% sucrose and loaded on top of a 300 µl cushion of 40% sucrose, then overlaid with 1.2ml 15% sucrose and 200 µl 5% sucrose. All sucrose solutions were at the same pH as the samples, and were wt/wt in TAN buffer at pH 8.0 or in MES buffer (50 mM MES, 100 mM NaCl) at pH 5.5. Gradients were centrifuged for 3 hr at 54,000 rpm at 4°C in a TLS55 rotor, and fractioned into the top 700 µl, middle 400 µl and bottom 1 ml. The ^3^H-cholesterol marker was quantitated by scintillation counting. 200 µl of each fraction were precipitated with 10% trichloroacetic acid and analyzed by SDS-PAGE and western blotting [29]. Purified human secreted placental alkaline phosphatase with a ST affinity tag (Seap) was used as a control protein [67], and was a kind gift from Yves Durocher, Biotechnology Research Institute, Montreal.

### Fusion-infection assay

The fusion-infection assay was performed essentially as described previously [41]. In brief, BHK cells grown on 96-well plates were washed twice with ice cold binding medium (RPMI without bicarbonate, 0.2% BSA, 10 mM Hepes, and 20 mM NH_4_Cl, pH 7.9). Virus stocks were diluted in binding medium and incubated with cells on ice for 3 h with gentle shaking. Cells were washed twice with binding medium to remove unbound virus and pulsed for 1 min at 37°C in 100 µl RPMI without bicarbonate, containing 0.2% BSA, 10 mM Hepes and 30 mM sodium succinate at pH 6.0 or 7.9, containing the indicated concentration of pr peptide. Infected cells were incubated in MEM plus 2% FCS and 50 mM NH_4_Cl for 4 h at 37°C, and then at 37°C for 2 d in the presence of 20 mM NH_4_Cl. The number of infected cells was quantitated by immunofluorescence using mouse polyclonal anti-DENV2 antibody. Infection observed at pH 7.9 represents virus that is endocytosed and fuses during 1 min at this pH.

### Generation of DENV1 E H244A mutant

The DENV1-WP infectious clone (reference [68], a kind gift from Dr. Barry Falgout) was digested with KpnI and a 3.3kb fragment including the E sequence was sub-cloned into the pGEM3Z vector to generate pGDENV1 3.3. pGDENV1 3.3 was used as a template to generate the E H244A mutation, using circular mutagenesis as previously described [69]. A 2.6kb BstB1/XhoI fragment containing the H244A mutation was sub-cloned into the DENV1-WP infectious clone to obtain DENV1-E H244A. The mutation was confirmed by restriction analysis and sequencing of the complete prM-E region. Two independent infectious clones were used to confirm the phenotype.

The WT and the mutant infectious clones were linearized by Sac II digestion and used as templates for in vitro transcription [70]. RNAs were electroporated into BHK cells and cells were cultured overnight at 37°C followed by 6 d at 28°C in MEM containing 2% FBS and 10 mM HEPES, pH 8.0. Progeny virus in the medium was quantitated by infectious center assay on indicator BHK cells, using mouse polyclonal anti-DENV2 antibody. To detect primary infection, aliquots of the electroporated cells were plated on coverslips, cultured 3 d at 37°C, and processed for immunofluorescence microscopy as above.

### Expression of prM-E and VLP production

WT and E H244A mutant DENV1 prM-E sequences were PCR-amplified from the pGDENV1 3.3 subclones described above, and cloned into pcDNA4/TO (Invitrogen). These constructs were transfected into T-REx™-293cells using Lipofectamine 2000 (Invitrogen) and selected in T-REx HEK medium containing 125 µg/ml Zeocin, all as previous described [61].

To test E protein folding and expression, 1×10^6^ WT and mutant E expressing cells were seeded in 10 cm plates, cultured for 24h, and then E protein expression was induced by culture for 36 h in 1.5 µg/ml tetracycline in DME medium with 10% FCS at 37°C. Cells were lysed in RIPA buffer (50 mM Tris-HCl pH 7.4, 150mM NaCl, 1% NP40, 0.5% Na-deoxycholate, 0.1% SDS, 1mM PMSF, 1× Roche complete protease inhibitor cocktail) on ice for 1 hr. The cell lysates were cleared by centrifugation for 30 min at 10,000×g and protein concentrations were quantitated and normalized. E proteins were immunoprecipitated from cell lysate samples (500 µg total cellular protein) using 20 µg purified mAb 4G2 or mAb 4E11 and 20 µl protein-G sepharose, or 30 µl Sango antibody and 20 µl protein-A sepharose. 4E11 and 4G2 immunoprecipitated samples were blotted with Sango. Sango immunoprecipitated samples were blotted with mouse anti DENV2 serum.

For VLP secretion studies, 2–3×10^6^ cells were seeded in 10 cm plates, cultured for 24h, and then induced by culture for 36 h in 1.5 µg/ml tetracycline in DME medium with 10% FCS at 37°C. The culture media were centrifuged at 10,000×g for 30 min to remove cell debris. VLPs were then pelleted through a 0.5 ml sucrose cushion by centrifugation at 54,000 rpm for 2 h at 4°C using a TLS55 rotor. To test the effect of neutralizing the pH of acidic cellular compartments, cells were seeded and induced as above. After 2 h of induction the media were changed to DME medium containing 20 mM HEPES pH 8.0, 2% FCS, and 1.5 µg/ml tetracycline plus 20 mM NH_4_Cl as indicated, and the incubation continued for a total of 36 h. E proteins in the cell lysates were immunoprecipitated using mAb 4G2. VLP and lysate samples were then analyzed by SDS-PAGE and western blot using Sango.

## Supporting Information

Figure S1 Open-book view of pr-E interface. Pr peptide is shown in cyan. DI, DII and DIII of E′ protein are colored red, yellow and blue, and the fusion loop at the DII tip is labeled. The important charged residues in the pr-E interface are numbered and shown as stick drawings in blue (positive) or red (negative). In this structure from DV2 16681, E′ residue 71 is a Glu, while the corresponding residue in NGC E′ protein is an Asp. Figure prepared from Protein Data Bank accession number 3C5X [34] using PyMOL.
(0.39 MB PDF)
