# Supplementary material for: In Vitro and In Vivo Studies Identify Important Features of Dengue Virus pr-E Protein Interactions
Source: PLoS Pathog. 2010 Oct 21;6(10):e1001157. doi: 10.1371/journal.ppat.1001157 (PMC2958806; doi:10.1371/journal.ppat.1001157)
Supplement: Figure S1 — Open-book view of pr-E interface. Pr peptide is shown in cyan. DI, DII and DIII of E′ protein are colored red, yellow and blue, and the fusion loop at the DII tip is labeled. The important charged residues in the pr-E interface are numbered and shown as stick drawings in blue (positive) or red (negative). In this structure from DV2 16681, E′ residue 71 is a Glu, while the corresponding residue in NGC E′ protein is an Asp. Figure prepared from Protein Data Bank accession number 3C5X [34] using PyMOL. (0.39 MB PDF) [file ppat.1001157.s001.pdf]

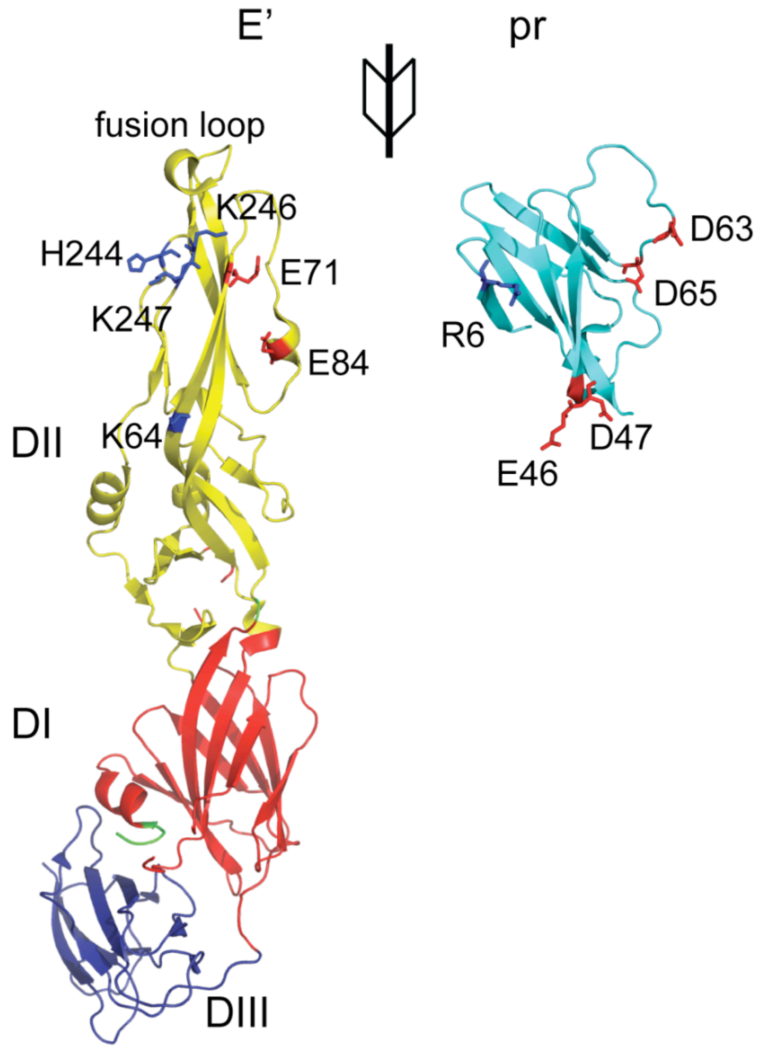

Figure S1. Open-book view of pr-E interface. Pr peptide is shown in cyan. DI, DII and DIII of E' protein are colored red, yellow and blue, and the fusion loop at the DII tip is labeled. The important charged residues in the pr-E interface are numbered and shown as stick drawings in blue (positive) or red (negative). In this structure from DV2 16681, E' residue 71 is a Glu, while the corresponding residue in NGC E' protein is an Asp. Figure prepared from Protein Data Bank accession number 3C5X [1] using PyMOL.

Reference:

1. Li L, Lok SM, Yu IM, Zhang Y, Kuhn RJ, et al. (2008) The flavivirus precursor membrane-envelope protein complex: structure and maturation. Science 319: 1830-1834.
